# Severe arsenic poisoning due to Ayurvedic supplements

**DOI:** 10.1002/ccr3.7733

**Published:** 2023-07-23

**Authors:** Jeremy Hardin, Justin Seltzer, Raymond Suhandynata, Benjamin Spiegel, Robin Silver, Diane Thomas, Henrik Galust, Nathan Friedman, Richard Clark, Jeremiah Momper

**Affiliations:** ^1^ Division of Medical Toxicology, Department of Emergency Medicine UC San Diego Health San Diego California USA; ^2^ VA San Diego Healthcare System San Diego California USA; ^3^ San Diego Division California Poison Control System San Diego California USA; ^4^ Department of Pathology UC San Diego Health San Diego California USA; ^5^ Skaggs School of Pharmacy and Pharmaceutical Sciences, UC San Diego Health San Diego California USA

**Keywords:** arsenic, ayurvedic supplements, heavy metal poisoning, lead

## Abstract

Patients that are taking Ayurvedic supplements have an increased risk of heavy metal toxicity. Lead, arsenic, and mercury are frequently identified in these supplements and can cause clinically significant toxicity. Clinicians should screen patients routinely for use of non‐pharmaceutical medications and supplements.

## Graphical abstract

Ayurvedic supplements containing heavy metals.

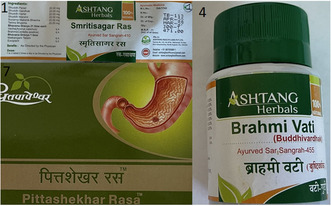

## 1 INTRODUCTION

Ayurveda, roughly translating to “the science of life,” is an over 2000‐year‐old traditional form of Indian medicine.^1^ The stated goal of Ayurveda is to maintain balance of the five bodily humors (water, earth, fire, air, and space) as well as the seven bodily juices (lymph, blood, muscle, fat, bone, marrow, and semen). Following various “detoxification” processes, the practice of *rasa shastra* combines herbs with metals, minerals, and gems in order to attempt to replete essential minerals in the body and rebalance the bodily humors.^2^, ^3^, ^4^ Consequently, heavy metals such as lead, arsenic, silver, cadmium, chromium, nickel, and mercury are commonly found in high amounts within Ayurvedic supplements due to both intentional addition and unintentional contamination.^1^, ^4^

Around half of the adult population in the United States uses dietary supplements (including homeopathic medicines) daily.^5^ There is a common misconception that because homeopathic/herbal medications are “natural” they are inherently nontoxic and safe.^2^ Around 20%–65% of Ayurvedic medicines available online contain detectable lead, mercury, and/or arsenic at levels exceeding standards for acceptable daily intake of toxic metals.^3^, ^4^ Under United States law dietary supplement manufacturers are not required to obtain premarket approval from the Food and Drug Administration (FDA).^5^ Manufacturers are not required to prove their product's health claims or even that it contains the ingredients they claim prior to bringing it to market. Dietary supplement surveillance is predominantly post‐market, after harm has already occurred, at which point the FDA can evaluate if it is unsafe, adulterated, or misbranded and potentially remove it from the market.^5^

We report a case of symptomatic arsenic and lead poisoning due to significant, long‐term exposure to prescribed Ayurvedic supplements. This report adds to the growing body of literature describing the dangers of unregulated Ayurvedic supplements in the United States.

## 2 MATERIALS AND METHODS

### 2.1 Participants

This is a single‐patient case report. Written consent was obtained per institutional policy. All diagnostic studies, including nerve conduction velocities, electromyography, and bone marrow biopsy, were performed using standard procedures and protocols. Interpretation of the results was provided by appropriately board‐certified clinicians.

### 2.2 Heavy metal testing

Quantitative determination of heavy metals present in the Ayurvedic supplements was performed via inductively coupled plasma mass spectrometry (ICP‐MS). Ten different supplements were tested for the presence of arsenic, mercury, cadmium, and lead. Each tablet was weighed, mechanically homogenized, then acid digested with concentrated nitric acid and hydrochloric acid until fully solubilized. The solutions were then serially diluted and analyzed along with quality control standards using an Agilent 7850 ICP‐MS with an active helium collision cell to prevent interference from argon chloride complexes that alter the measurement of arsenic. All quality controls were measured within 20% of their target values. The amount of arsenic, mercury, cadmium, and total lead in each tablet was determined by taking the measured concentration of each dilution, multiplying by the dilution factor of the sample, and multiplying by the weight of each individual tablet.

## 3 RESULTS

### 3.1 Case report

**FIGURE 1:**
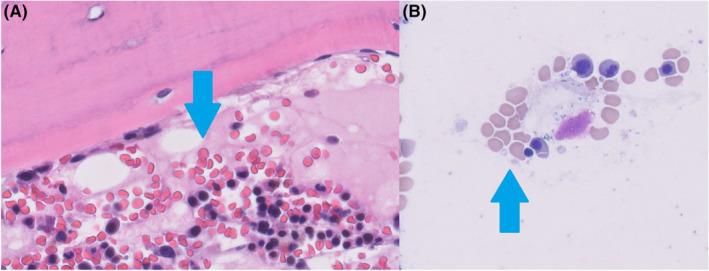
Bone marrow biopsy obtained from the right posterior iliac crest. There are features of erythroid hyperplasia (A), erythroid dysplasia (B), reticulocytosis, and hypolobated megakaryocytes. Ringed sideroblasts and basophilic stippling were not seen. Biomarker studies including cytogenetics, fluorescence in situ hybridization, and an extensive molecular gene mutation panel showed no evidence of any clonal myeloid processes. There was also no evidence of plasma cell lymphoma, lymphoproliferative disorder, or infiltrative marrow processes.

A 75‐year‐old Caucasian female with a past medical history of postural tachycardia syndrome presented to the emergency department with fatigue and decreased sensation in her distal extremities. She was found to have pancytopenia and was discharged with referral for outpatient hematology evaluation. Hematology obtained a bone marrow biopsy, which demonstrated erythroid hyperplasia, erythroid dysplasia, reticulocytosis, as well as small hypolobated megakaryocytes (Figure 1A,B), and recommended formal neurologic testing.

**FIGURE 2:**
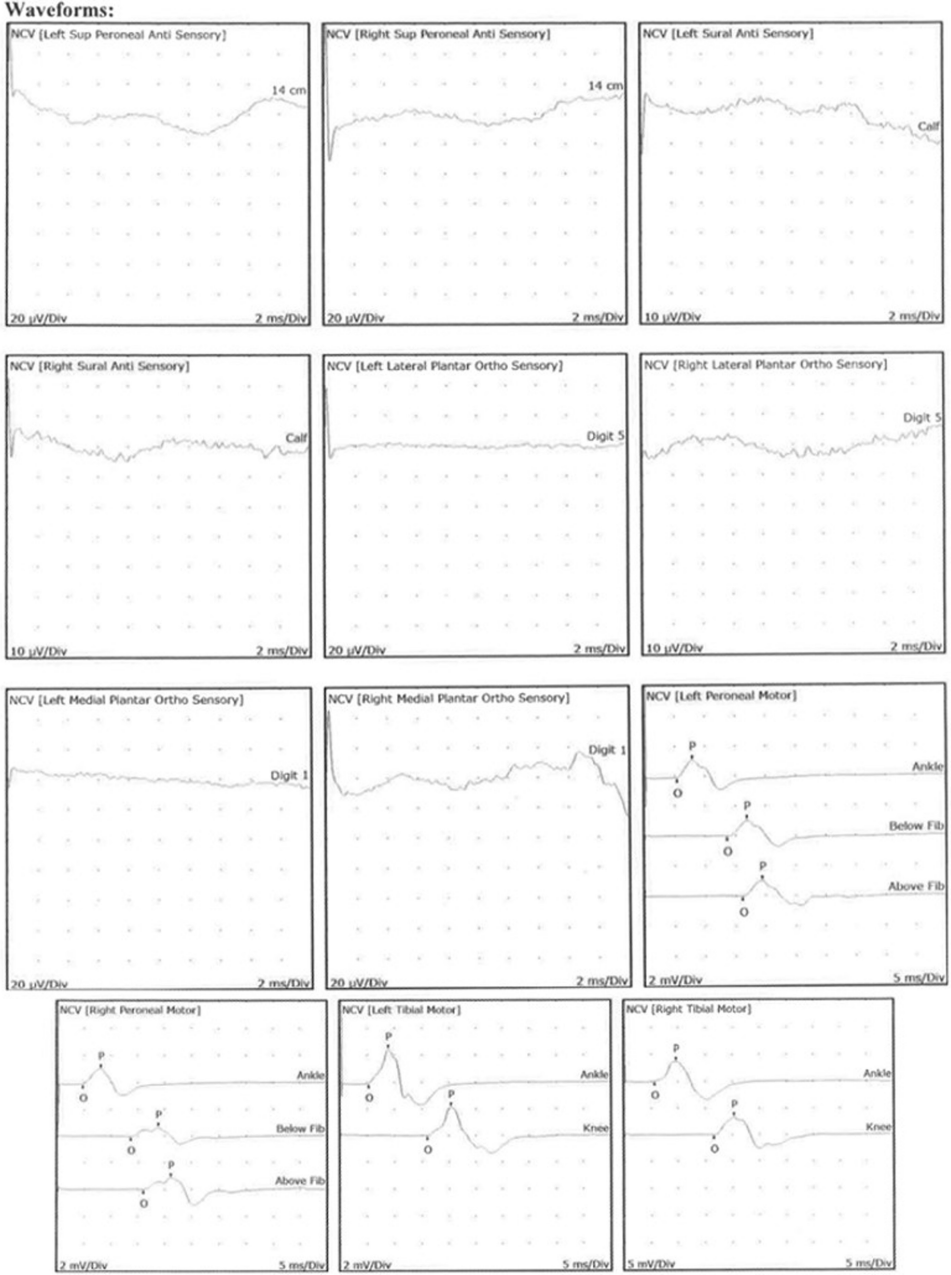
Nerve conduction velocities and electromyograph demonstrated evidence of small fiber polyneuropathy with distal degeneration of sensory greater than motor axons.

Neurologic evaluation revealed normal motor strength, diminished deep tendon reflexes without clonus or spasticity, and normal gait. Nerve conduction velocities and electromyography were found to be abnormal (Figure 2). Based on these findings, the neurologist then obtained heavy metal testing that showed elevated blood lead (26.4 μg/dL, normal <10 μg/dL) and arsenic (140.6 μg/L, normal <50 μg/L) concentrations as well as elevated urine arsenic (total 871.3 μg/L; inorganic 1683.8 μg/L, methylated 1730.4 μg/L, and organic <5 μg/L) and lead (21.2 μg/L) concentrations. These results prompted medical toxicology referral.

The patient stated that she has been prescribed various Ayurvedic supplements by a holistic energy healer for “many years” due to an imbalance of her bodily humors toward the vata (air) element causing anxiety, inability to gain weight, anemia, palpitations, and heartburn. She endorsed taking approximately 50 tablets per day. She received her most recent batch of supplements from India 6 weeks prior to developing symptoms and had been abstinent for 1 month at the time of toxicology evaluation. Apart from these supplements, she had no lifestyle, occupational, or dietary risk factors for heavy metal exposure.

Repeat blood levels 1 month after discontinuation demonstrated decreasing blood arsenic (13.6 μg/L) and stable blood lead (27.7 μg/dL) concentrations. Testing of a 24‐h urine collection demonstrated a lead concentration of 24.1 μg/L and a methylated arsenic level of 37.8 μg/L with undetectable inorganic and organic arsenic. Repeat complete blood counts demonstrated resolution of her pancytopenia. Due to the patient's decreasing heavy metal concentrations and plan to continue abstaining from Ayurvedic supplements, chelation was deferred in favor of close outpatient monitoring. At 135 days follow‐up, the patient reported that her weakness had improved but her distal extremity sensation remained diminished and unchanged. Repeat blood testing at that time revealed an undetectable blood arsenic concentration and a blood lead concentration of 28 μg/dL.

### 3.2 Source analysis findings

**FIGURE 3:**
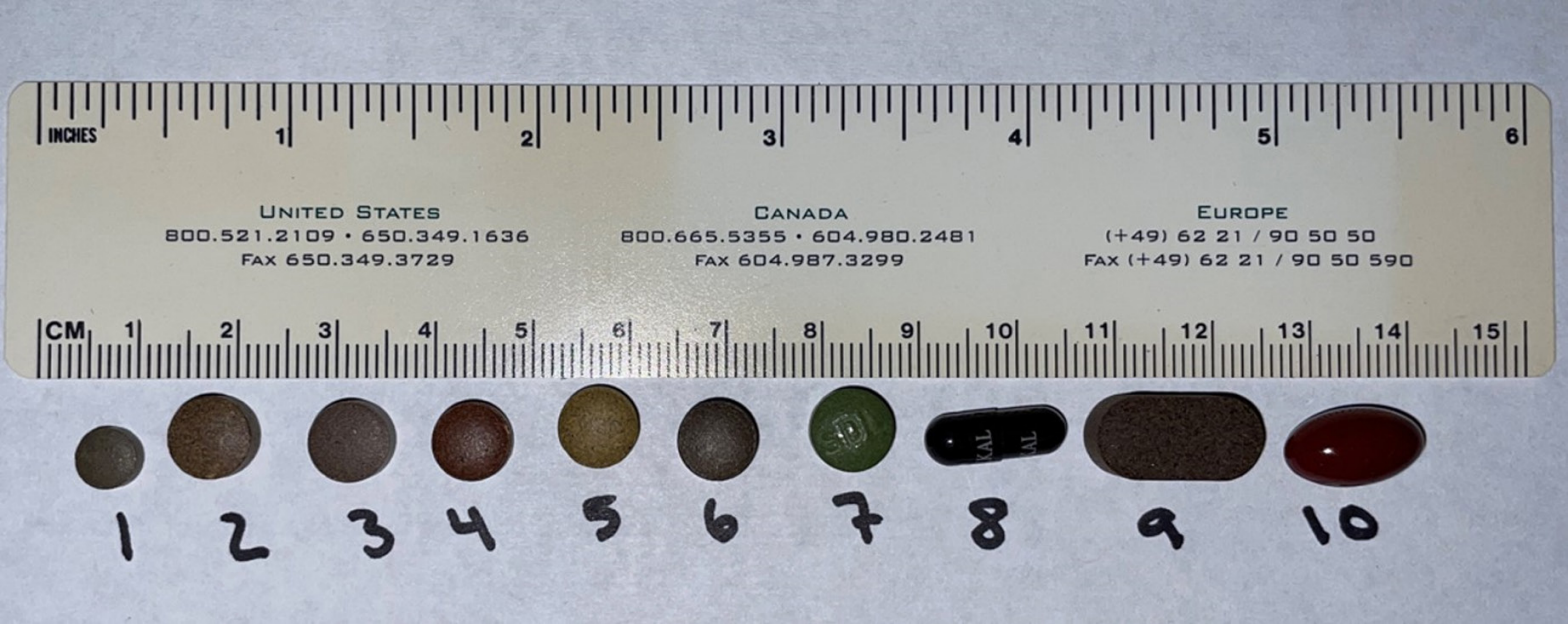
The 10 Ayurvedic supplements that were being taken by the patient.

**FIGURE 4:**
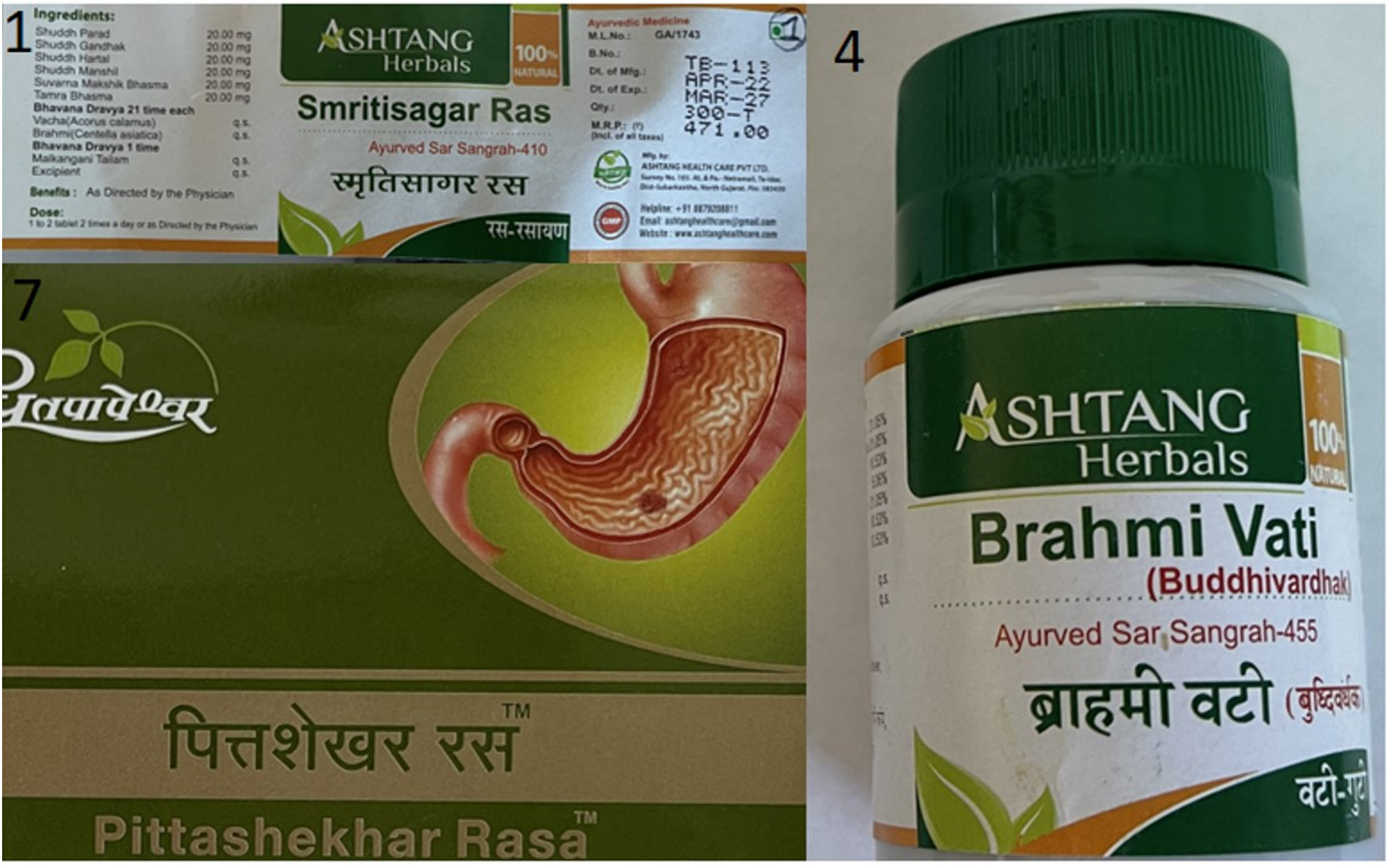
The Ayurvedic supplements (#1, 4, 7) identified as containing toxic amounts of heavy metals.

All 10 Ayurvedic supplements being taken by the patient were tested with ICP‐MS (Figure 3). All tablets contained measurable heavy metals. Eight out of 10 contained arsenic, 9 out of 10 contained mercury, and 7 out of 10 contained lead (Table 1). Three supplements (#1, 4, 7) had heavy metal concentrations, a magnitude higher than the others with supplement #1 being composed of 9.3% arsenic by weight (Figure 4). Daily heavy metal doses were calculated based off the patient's reported daily supplement consumption and revealed a daily arsenic dose of greater than 650 mg/day and a mercury dose of greater than 300 mg/day.

**TABLE 1:** Results of Ayurvedic supplement analysis via inductively coupled plasma mass spectrometry.

Medicine ID	As (mg)	Hg (mg)	Pb (mg)	Recommended daily dose	Tablets taken by patient	Daily as dose (mg)	Daily Hg dose (mg)	Daily Pb dose (mg)
1	16.34	6.12	<LOQ	4	40	653.6	244.8	<0.1
4	0.90	18.64	<LOQ	4	4	3.62	74.58	<0.01
7	<LOQ	0.64	<LOQ	4	1	<0.003	0.64	<0.003
	As (μg)	Hg (μg)	Pb (μg)			As (μg)	Hg (μg)	Pb (μg)
2	0.40	6.39	0.25	6	10	4.04	63.94	2.52
3	1.15	9.10	1.82	6	4	4.61	36.42	7.26
5	0.24	0.95	0.16	6	2	0.49	1.9	0.32
6	1.19	4.17	0.80	6	4	4.74	16.67	3.2
8	0.21	0.68	0.34	2	4	0.82	2.71	1.35
9	0.19	0.10	0.15	6	4	0.74	0.39	0.62
10	<LOQ	<LOQ	0.04	4	4	<0.1	<0.1	0.15

## 4 DISCUSSION

It is well known that Ayurvedic supplements can contain heavy metals. Often this is intentional as the manufacturing process involves the elaborate preparation of metals including cinnabar (mercuric sulfide), galena (lead sulfide), realgar (arsenic sulfide), and white arsenic (arsenic trioxide).^6^, ^7^ Multiple of this patient's Ayurvedic supplements were found to contain toxic levels of both arsenic and mercury, as well as trace amounts of lead. This is consistent with previously published literature looking broadly at Ayurvedic supplements available in the United States as well as the practice of *rasa shastra* specifically.^4^, ^5^, ^8^ Of note, supplement #1 was labeled to contain 20 mg “shuddh hartal” which translates to “pure arsenic” and was composed of 16 mg arsenic per tablet. At the time of writing this article, Ayurvedic supplements advertising the presence of arsenic trioxide were easily available to order online and ship anywhere within the United States.

Around half of the adult population in the United States uses dietary supplements and herbal preparations daily.^9^ It is commonly thought among the lay population that remedies labeled as “natural” or “herbal” are inherently nontoxic and safe.^3^ However, around 20%–65% of Ayurvedic herbal supplements tested have been found to contain detectable lead, mercury, and/or arsenic at levels exceeding standards for acceptable daily intake.^4^, ^5^ Unfortunately, in the United States there is little required dietary supplement safety and quality testing. Most surveillance is performed in the post‐marketing setting, after harm has already occurred, at which point the FDA can remove products from the market that are found to be unsafe, adulterated, or misbranded.^5^ However, supplements and herbs obtained from outside the United States, as in this case, can be even less regulated and difficult to monitor.^8^

Of the detected heavy metals, the patient's elevated arsenic level is of the highest clinical concern. The toxicity of arsenic is well established and known both historically and by modern report. It is odorless, tasteless, and highly toxic.^10^ The mechanism of toxicity is thought to be multifactorial but is currently unknown; possible mechanisms include the generation of reactive oxidation species via the reaction of trivalent arsenic compounds with sulfur and substitution of arsenic for phosphate in various biochemical reactions. Inorganic arsenic, which is the form our patient ingested, is dramatically more toxic than the organic form, which is commonly found in seafood. Once ingested, arsenic is hepatically metabolized to methylated arsenic species that can then be eliminated.^10^ Our patient's initial urine speciation is consistent with inorganic arsenic exposure given high levels of detected inorganic and methylated species with undetectable organic arsenic.

Our patient presented with findings consistent with arsenic neurotoxicity, namely peripheral neuropathy with sensory greater than motor deficits. These deficits can worsen over weeks despite the withdrawal of the source via a phenomenon known as “coasting.”^11^, ^12^ As was seen in this case, bone marrow biopsy can reveal hypercellularity and erythroid hyperplasia. Basophilic stippling and Howell–Jolly bodies can also be present.^13^ Other classic findings not present in this case include skin pigmentation changes, plantar/palmar hyperkeratosis, and Mee's lines.^14^ Symptomatic patients can be treated with a variety of chelating agents, including D‐penicillamine, dimercaprol, dimercaptosuccinic acid, and dimercaptopanesulfonic acid, though arsenic neurotoxicity responds poorly to chelation.^14^ Chelation was deferred in this case as our patient was clinically improving with abstinence and it was unlikely to improve her neurotoxic symptoms.

The patient's elevated blood lead concentrations may have contributed to her illness but these elevations were mild when compared to her arsenic exposure and her clinical findings were less consistent. Chronic lead exposure has been shown to cause a predominantly motor neuropathy secondary to axonal degeneration which was not seen in this patient.^15^ Similarly, a blood lead level of 55 μg/dL is associated with clinically significant heme biosynthesis inhibition and anemia—a level nearly double what was present in our patient.^16^ We speculate that the stability of her blood lead levels is due to slow redistribution from the tissues following extensive chronic exposure.

While multiple of her supplements contained significant amounts of mercury, the rate of absorption of elemental mercury through an intact gastrointestinal tract following oral ingestion is extremely low and clinically unimportant with regards to causing systemic toxicity.^17^ Furthermore, she had multiple blood and urine samples without detectable mercury concentrations.

This case is limited primarily by lack of environmental testing of the home and water. These were not performed due to the prohibitive cost, lack of identifiable risk factors for environmental exposure, and previously suspected potential sources. While our suspicion is low for an environmental or alternative source, we cannot entirely exclude this possibility. Our analysis is also limited by sample quality. As only a sample of each supplement could be tested due to cost constraints, underestimation or overestimation of heavy metal content is possible, especially given known variability in composition.^3^

## 5 CONCLUSION

In summary, we present a case of clinically significant arsenic and lead poisoning secondary to Ayurvedic supplements. This patient had an extensive workup that revealed many of the classic signs and symptoms of chronic arsenic toxicity. Laboratory testing confirmed the presence of these heavy metals in Ayurvedic supplements the patient was known to be taking. Clinicians should caution patients about the known risks of Ayurvedic supplement heavy metal toxicity, particularly with *rasa shastra* preparations, and screen patients routinely for use of non‐pharmaceutical medications and supplements.

## AUTHOR CONTRIBUTIONS

**Jeremy Hardin:** Conceptualization; data curation; formal analysis; investigation; methodology; visualization; writing – original draft; writing – review and editing. **Justin Seltzer:** Conceptualization; formal analysis; methodology; writing – original draft; writing – review and editing. **Raymond Suhandynata:** Data curation; formal analysis; investigation; methodology; resources; validation; writing – review and editing. **Benjamin Spiegel:** Formal analysis; investigation; methodology; writing – review and editing. **Robin Silver:** Formal analysis; investigation; methodology; writing – review and editing. **Diane Thomas:** Formal analysis; investigation; methodology; writing – review and editing. **Henrik Galust:** Conceptualization; data curation; formal analysis; methodology; visualization; writing – review and editing. **Nathan Friedman:** Conceptualization; data curation; formal analysis; writing – review and editing. **Richard Clark:** Conceptualization; data curation; formal analysis; investigation; supervision; visualization; writing – review and editing. **Jeremiah Momper:** Conceptualization; data curation; formal analysis; investigation; methodology; project administration; supervision; validation; visualization; writing – review and editing.

## FUNDING INFORMATION

No funding was secured for this study.

## CONFLICT OF INTEREST STATEMENT

All authors have no conflicts of interest to disclose.

## CONSENT

The patient provided informed written consent for this research per our institutional guidelines.

## Data Availability

Supplement analysis data can be provided upon request.

## References

[1] Treleaven J , Meller S , Farmer P , Birchall D , Goldman J , Piller G . Arsenic and ayurveda. Leuk Lymphoma. 1993;10(4–5):343‐345. 7693104 10.3109/10428199309148558

[2] Lynch E , Braithwaite R . A review of the clinical and toxicological aspects of 'traditional' (herbal) medicines adulterated with heavy metals. Expert Opin Drug Saf. 2005;4(4):769‐778. 16011453 10.1517/14740338.4.4.769

[3] Saper RB , Phillips RS , Sehgal A , et al. Lead, mercury, and arsenic in US‐ and Indian‐manufactured ayurvedic medicines sold via the internet. JAMA. 2008;300(8):915‐923. 18728265 10.1001/jama.300.8.915 PMC2755247

[4] Mikulski MA , Wichman MD , Simmons DL , Pham AN , Clottey V , Fuortes LJ . Toxic metals in ayurvedic preparations from a public health lead poisoning cluster investigation. Int J Occup Environ Health. 2017;23(3):187‐192. 29528276 10.1080/10773525.2018.1447880 PMC6060866

[5] Dwyer JT , Coates PM . Why Americans need information on dietary supplements. J Nutr. 2018;148:1401S‐1405S. 31505678 10.1093/jn/nxy081 PMC6857605

[6] Satpute AD . Rasa Ratna Samuchaya of Vagbhatta. Chaukhamba Sanskrit Pratishtana; 2003:5.

[7] Shastri K . Rasa Tarangini of Sadananda Sharma. Motilal Banarsidas; 1979.

[8] Khandpur S , Malhotra AK , Bhatia V , et al. Chronic arsenic toxicity from ayurvedic medicines. Int J Dermatol. 2008;47(6):618‐621. 18477160 10.1111/j.1365-4632.2008.03475.x

[9] Nutrition Business Journal . Supplement Business Report 2017. New York: Penton Media 2017.

[10] Hughes MF , Beck BD , Chen Y , Lewis AS , Thomas DJ . Arsenic exposure and toxicology: a historical perspective. Toxicol Sci. 2011;123(2):305‐332. 21750349 10.1093/toxsci/kfr184 PMC3179678

[11] Mochizuki H . Arsenic neurotoxicity in humans. Int J Mol Sci. 2019;20(14):3418. 31336801 10.3390/ijms20143418 PMC6678206

[12] Toledano M . Toxin‐induced neuropathies. Neurol Clin. 2020;38(4):749‐763. 33040859 10.1016/j.ncl.2020.06.002

[13] Feussner JR , Shelburne JD , Bredehoeft S , Cohen HJ . Arsenic‐induced bone marrow toxicity: ultrastructural and electron‐probe analysis. Blood. 1979;53(5):820‐827. 435641

[14] Hall AH . Chronic arsenic poisoning. Toxicol Lett. 2002;128(1–3):69‐72. 11869818 10.1016/s0378-4274(01)00534-3

[15] Wu PB , Kingery WS , Date ES . An EMG case report of lead neuropathy 19 years after a shotgun injury. Muscle Nerve. 1995;18(3):326‐329. 7870111 10.1002/mus.880180310

[16] Mitra P , Sharma S , Purohit P , Sharma P . Clinical and molecular aspects of lead toxicity: an update. Crit Rev Clin Lab Sci. 2017;54(7‐8):506‐528. 29214886 10.1080/10408363.2017.1408562

[17] Agency for Toxic Substances and Disease Registry‐ATSDR . Public health service. Toxicological Profile for Mercury. US Department of Health and Human Services; 1999:1‐676.

